# 45S rDNA external transcribed spacer organization reveals new phylogenetic relationships in *Avena* genus

**DOI:** 10.1371/journal.pone.0176170

**Published:** 2017-04-27

**Authors:** Joana Rodrigues, Wanda Viegas, Manuela Silva

**Affiliations:** Linking Landscape, Environment, Agriculture and Food (LEAF), Instituto Superior de Agronomia, Universidade de Lisboa, Lisboa, Portugal; Consiglio Nazionale delle Ricerche, ITALY

## Abstract

The genus *Avena* comprises four distinct genomes organized in diploid (AA or CC), tetraploid (AABB or AACC) and hexaploid species (AACCDD), constituting an interesting model for phylogenetic analysis. The aim of this work was to characterize 45S rDNA intergenic spacer (IGS) variability in distinct species representative of *Avena* genome diversity–*A*. *strigosa* (AA), *A*. *ventricosa* (CvCv), *A*. *eriantha* (CpCp), *A*. *barbata* (AABB), *A*. *murphyi* (AACC), *A*. *sativa* (AACCDD) and *A*. *sterilis* (AACCDD) through the assessment of the 5’ external transcribed spacer (5’-ETS), a promising IGS region for phylogenetic studies poorly studied in *Avena* genus. In this work, IGS length polymorphisms were detected mainly due to distinct 5’-ETS sequence types resulting from major differences in the number and organization of repeated motifs. Although species with A genome revealed a 5’-ETS organization (A-organization) similar to the one previously described in *A*. *sativa*, a distinct organization was unraveled in C genome diploid species (C-organization). Interestingly, such new organization presents a higher similarity with other Poaceae species than A-genome sequences, supporting the hypothesis of C-genome being the ancestral *Avena* genome. Additionally, polyploid species with both genomes mainly retain the A-genome 5’-ETS organization, confirming the preferential elimination of C-genome sequences in *Avena* polyploid species. Moreover, 5’-ETS sequences phylogenetic analysis consistently clustered the species studied according to ploidy and genomic constitution supporting the use of ribosomal genes to highlight *Avena* species evolutive pathways.

## Introduction

Oat (*Avena* L., Poaceae family) is one of the most cultivated cereals worldwide and a valuable resource in several countries both for human consumption and animal feed [1]. Genus *Avena* L. constitutes an interesting model for phylogenetic studies comprising 26 species with different genomes and ploidy levels [2]. Within this genus, the basic number of chromosomes is x = 7 and there are diploid species with AA or CC genomes; tetraploids with AABB or AACC genomes and hexaploids with AACCDD genome [3, 4]. While the C-genome is quite different from the other ones [5], B and D genomes are very similar to the A-genome [4, 6]. Besides, since diploid species with B or D genomes have not been found, it seems that both diverged from an ancestral A' genome [7]. The model proposed for hexaploid species evolution involves the hybridization of two diploid species, followed by chromosome duplication, originating an AACC ancient genome. Afterwards, this allotetraploid underwent another hybridization event with an AA diploid species and subsequent chromosome duplication. The evolution of the tetraploid species with AABB genomes was suggested to be an unrelated event that followed a diploid AA species autopolyploidization (for a review see [8]). Cytogenetic evaluation of ribosomal *loci* has been extensively used to study *Avena* species variability [4, 9–12] suggesting that allopolyploidization was followed by a preferential decrease in the number and/or size of C-genome NORs (nucleolar organizing regions) [5].

To clarify *Avena* diversity and evolution several molecular markers have been used such as simple sequence repeats (SSR) or microsatellites [13–16]; restriction fragment length polymorphism (RFLP) [17, 18]; amplified fragment length polymorphism (AFLP) [19] and inter-retrotransposon amplified polymorphism (IRAP), retrotransposon-microsatellite amplified polymorphism (REMAP) and Inter Simple Sequence Repeat (ISSR) [12, 20, 21]. 45S rDNA units, organized in tandem repeats of IGS-18S-ITS1-5.8S-ITS2-25S [22], have also been targeted for *Avena* phylogenetic analysis. The internal transcribed spacers (ITS1 and ITS2) and the ITS1-5.8S-ITS2 region have been studied in detail in *Avena* species [5, 12, 23–25]. Additionally, Polanco and De La Vega [26] detected intergenic spacer (IGS) length polymorphism in *A*. *sativa* cv. Cometa and the analysis of the longest sequence (4098bp, Accession Number: X74820.1) disclosed five different types of repeats—A, B, C, D and E—being each one present in two to 13 repetitions. These results were latter confirmed through PCR amplification in an extensive collection of *A*. *sativa* accessions [27]. Inter and intraspecific variations in the IGS have been also described in *Avena* by RFLP [23, 28, 29]. Nikoloudakis and colleagues [23] assessed IGS diversity through restriction enzyme digestion of a ~4000bp fragment amplified from fifty four accessions belonging to 23 *Avena* taxa and found 150 different banding patterns. Within the IGS, the assessment of the 5' external transcribed spacer (5’-ETS) has also been used to clarify phylogenetic relationships and differentiate between closely related species of distinct genera of Asteraceae [30] and Fagaceae [31]. However, this particular IGS region that begins with the transcription initiation site (TIS) and encompasses several pre-rRNA processing signals [26] was never detailed studied in *Avena* species.

The present work aimed to evaluate rDNA intergenic sequences diversity in several *Avena* species with distinct ploidy levels and genome compositions contributing to enlighten *Avena* genus evolution pathways.

## Materials and methods

The plant material used includes the following species: *A*. *strigosa* (genome AA, 5284) obtained from EAN Germplasm Bank (Oeiras, Portugal, PRT005); *A*. *ventricosa* (genome CvCv, PI657337); *A*. *eriantha* (genome CpCp, Clav9050); *A*. *barbata* (genome AABB, PI367338); *A*. *murphyi* (genome AACC, PI657606); *A*. *sativa* (genome AACCDD, Clav8250) and *A*. *sterilis* (genome AACCDD, PI267989) obtained from United States Department of Agriculture, Agricultural Research Service (USDA). At least three seeds were germinated and plants were maintained in growth chambers at 16h/day (22*°*C) and 8h/night (15*°*C). Leaves from different one month old plants were stored at -80*°*C for further utilization and plants were then maintained in greenhouse until life cycle completion.

### DNA extraction and PCR experiments

**Fig 1:**
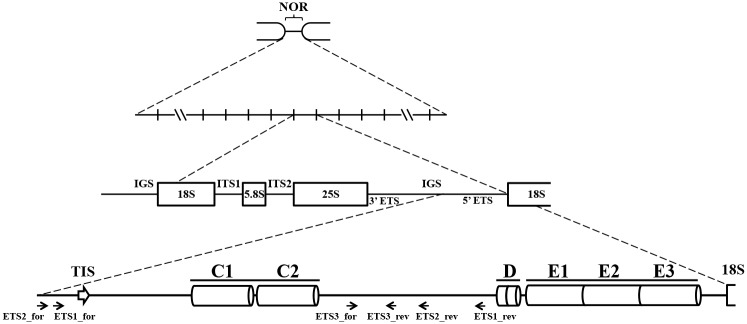
Organization of 45S rDNA units in *A*. *sativa*. IGS—intergenic spacer; 3’-ETS– 3’ External transcribed spacer; TIS—transcription initiation site; 5’-ETS– 5’ external transcribed; ITS—internal transcribed spacers. ETS1, ETS2 and ETS3 primers used for intergenic sequences amplification designed based on the *A*. *sativa* sequence (Accession Number: X74820.1).

DNA extractions were performed with DNA Cell & Tissue Kit (Citogene) according to the manufacturer’s instructions. DNA was quantified and stored at -20*°*C. Primers used are listed in Table 1 and their schematic representation is presented in Fig 1 in relation to *A*. *sativa* rDNA spacer published sequence (Accession Number: X74820.1). PCR experiments were performed in at least three replicates for each primer combination in a 20 μl volume with: 1x PCR buffer; 1.5 mM MgCl_2_; 0.1 mM dNTP’s; 0.25 to 1 μM of each primer; 1 U NZYTaq DNA polymerase (NZYtech); 30 to 50 ng DNA template and Milli-Q H_2_O for the remaining volume. The IGS was amplified using the modified touchdown PCR program [32]: 94°C for 4 minutes; 10 cycles of 94°C for 1 minute; 65°C for 30 seconds, decreasing 0.5°C each cycle; 72°C for 4 minutes; 25 cycles of 94°C for 1 minute; 60°C for 30 seconds; 72°C for 4 minutes and 10 minutes of final extension at 72°C. Amplification of partial 5’-ETS sequences was carried as followed: 94°C for 4 minutes; 30 cycles of 94°C for 1 minute; 57°C for 45 seconds; 72°C for 90 seconds; with 15 minutes of final extension at 72°C. The complete 5’-ETS sequences were amplified with the same program but with 2 minutes in each extension step. The amplification of the ITS2 region used as control was performed with the partial 5’-ETS program but with 60°C in the annealing step. PCR products were separated in 1 or 1.7% agarose gels with 1 Kb Plus DNA Ladder (Invitrogen) as molecular marker. Gels were detected with ethidium bromide and photographed using BIO-Rad GEL DOC 2000.

**Table 1:** Primers used to amplify 45S rDNA sequences.

Primer designation	Sequence
**25S**	5’–GACGACTTAAATACGCGACGG– 3’
**18S**	5’–AGACAAGCATATGACTACTGG– 3’
**ETS1_for**	5’–TGTACCCCTCCTTCACAAGC– 3’
**ETS1_rev**	5’–CGAGGCTTCCTTGATAGCAC– 3’
**ETS2_for**	5’–AAAACCCGTGCAGGAACTC– 3’
**ETS2_rev**	5’–CAAGCACTTGAAAGGCAACA– 3’
**ETS3_for**	5’–GGACACTCAGCACGCCTTC– 3’
**ETS3_rev**	5’–ACACGGGTCCAAAGCTACTC– 3’
**ETS4_for**	5’–TCGGTGTTTACATGTTCGAG– 3’
**ITS_for**	5’–GCATCGATGAAGAACGCAGC– 3’
**ITS_rev**	5’–TCCTCCGCTTATTGATATGC– 3’

Notes:

Primers were designed using Primer3: ETS1_for and rev, ETS2_for and rev and ETS3_for and rev based on the *A*. *sativa* rDNA spacer published sequence (Accession Number: X74820.1); ETS4_for based on the *A*. *ventricosa* partial sequences obtained in this work (Accession Number: KM586761); and 18S based on the *T*. *aestivum* 18S sequence (Accession Number: X07841). Primer 25S was designed by [26] and primers ITS_for and ITS_rev were designed by [33] (ITS3 and ITS4, respectively).

Firstly, primers 25S and 18S were used to amplify the IGS sequences. Primers ETS1_for and rev, ETS2_for and rev were used to obtain 5’-ETS partial sequences (between TIS sequence and D repeat). From those, primer ETS1_for was chosen to obtain the entire 5’-ETS in combination with 18S primer. Primers ETS3_for and rev were used combined with other primers to further confirm the entire 5’-ETS sequencing and ETS4_for primer, designed based on the *A*. *ventricosa* partial sequences obtained in this work (Accession Number: KM586761), was used to assess the occurrence of C-organization sequences. Finally, ITS_for and rev primers were used as internal controls to amplify the 45S rDNA ITS2 sequences.

### Sequence cloning

Selected bands were isolated from agarose gels, purified with NZYGelpure (NZYtech) and the fragments obtained were ligated into the pCR2.1 vector (Invitrogen) cloned into NZY5α Competent cells (NZYtech). Transformed cells were incubated at 37*°*C overnight on LB agar plates containing ampicillin (100 μg/ml) and X-Gal (20 μg/ml) and colony screening was performed by PCR with M13 primers. Selected colonies were grown overnight at 37*°*C, 250 rpm in liquid LB medium with ampicillin. Plasmids were purified with NZY Miniprep (NZYtech) and the inserted fragments were sequenced through the Sanger method (Stabvida). The accession numbers of all sequences obtained (Accession Numbers: KM586737 to KM586775) are listed in S1 Table.

### Sequence and phylogenetic analysis

The phylogenetic analysis was tested by aligning the sequences using different methods with the default settings: Clustal Omega, MUSCLE, MAFFT and T-COFFEE (http://www.ebi.ac.uk/Tools/msa/). Next, the best evolutionary model was tested in JModelTest2 with 3 substitutions schemes [34]. A similar tool is available in MEGA version 6 [35] and was also tested. The best phylogenetic model was selected based on the lowest BIC (Bayesian Information Criterion) score and in general the same models had low scores in both programs. Lastly, the phylogenetic trees for the different alignments and with the different models were made in MEGA6 and in MrBayes [36] and all trees generated clustered the species similarly. Thus, the phylogenetic analysis was performed in MEGA version 6 and the sequences were aligned using the MUSCLE application with default settings. The best phylogenetic model was tested and selected based on the lowest BIC score and the Maximum Likelihood trees with 1000 bootstrap repetitions were obtained. Since some sequences presented insertions, one standard sequence of each species with the highest similarity with the published sequence for A. *sativa* (Accession Number: X74820.1) was used to construct the tree based on the complete 5’-ETS sequences. Similar phylogenetic trees were built using the 5’-ETS sequences excluding all repeat motifs identified.

The alignment of all sequences in S2 Fig was obtained using the ClustalW application and manually adjusted using BioEdit Sequence Alignment Editor version 7.2.5 [37]. Dotplots to identify repeats were constructed in Unipro UGENE [38] with minimum repeat length of 8bp and 100% identity.

## Results

**Fig 2:**
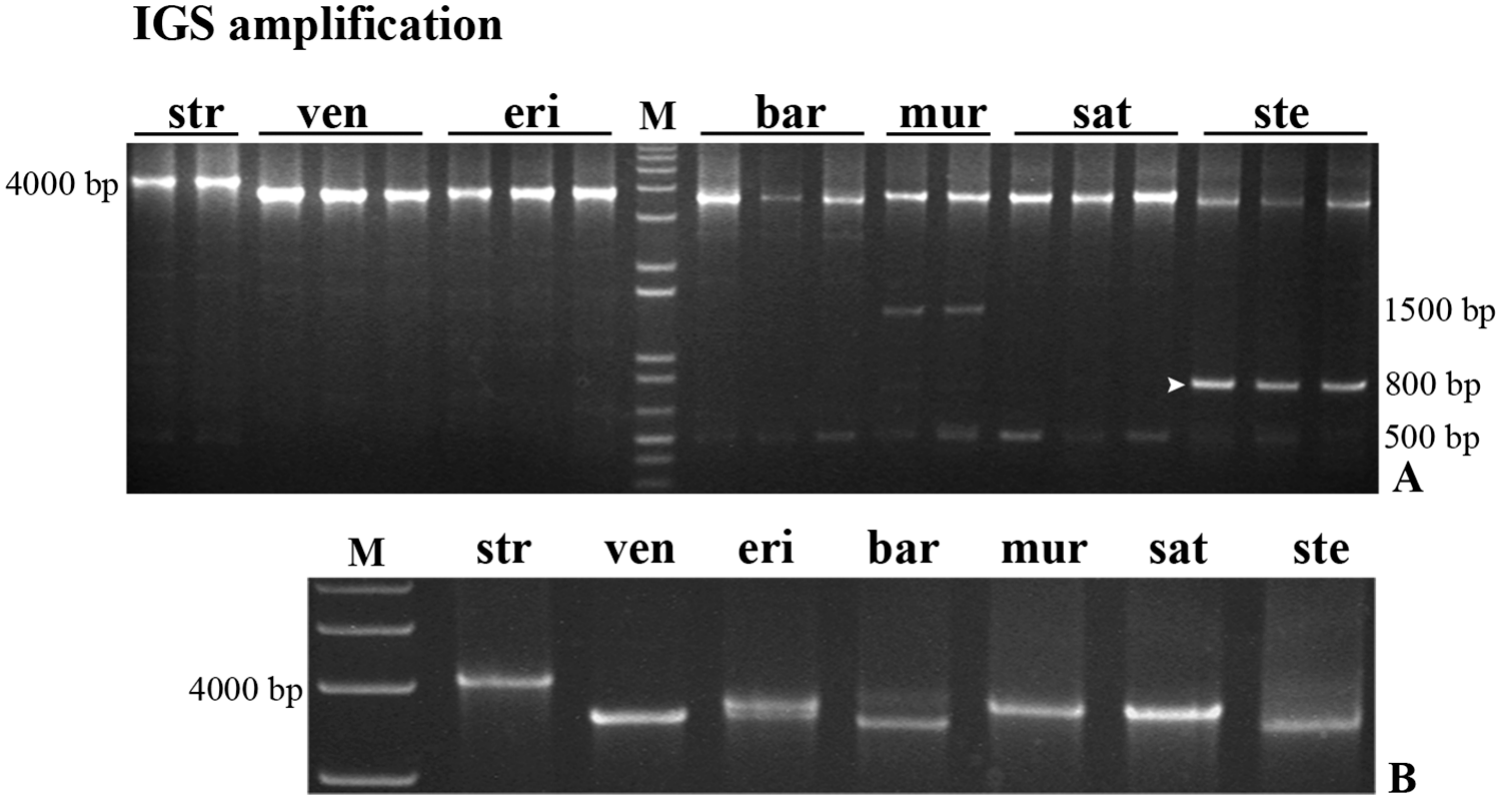
IGS complete sequence. PCR amplification of the complete IGS sequence from *A*. *strigosa* (str); *A*. *ventricosa* (ven); *A*. *eriantha* (eri); *A*. *barbata* (bar); *A*. *murphyi* (mur); *A*. *sativa* (sat); and *A*. *sterilis* (ste) using primers 25S and 18S. **(A)** Amplification from different individuals of each species. **(B)** Bands with the size approximate to the expected from *A*. *sativa* IGS sequence. M: molecular marker 1Kb+. The arrowhead indicates the ~800bp *A*. *sterilis* band sequenced.

The amplification of the entire 45S rDNA intergenic spacer (IGS) using 25S and 18S primers from different individuals of each species did not reveal intraspecific diversity (Fig 2A). A major band with a size approximated to the expected from the *A*. *sativa* rDNA IGS published sequence (4098 bp, Accession Number X74820.1) was observed in all species analyzed. Yet, a clear interspecific IGS length polymorphism was detected (~4000bp gel region, Fig 2B) with *A*. *strigosa* presenting the longest spacer with ~4000bp, and *A*. *ventricosa*, *A*. *barbata* and *A*. *sterilis* showing smaller IGSs with similar dimension (~3600bp), as well as *A*. *eriantha*, *A*. *murphyi* and *A*. *sativa* (~ 3800bp). Moreover, an additional band with lower intensity can be seen in this gel region in *A*. *eriantha* and A. *barbata*.

Additionally, bands with much shorter dimension than the ~4000bp expected were detected (Fig 2A). From them a band with ~500bp was observed in most species except in *A*. *vetricosa* and *A*. *eriantha* and an additional band with ~1500bp was observed in *A*. *murphyi*. In *A*. *sterilis* a smaller band with ~800bp, presenting higher intensity than the ~4000bp band, was gel isolated and sequenced. This sequence corresponds to a truncated shorter variant of the IGS possibly nonfunctional due to the lack of transcription initiation site (for detail see S1 Fig).

### 5’-ETS sequences analysis unraveled marked differences between A and C genomes

**Fig 3:**
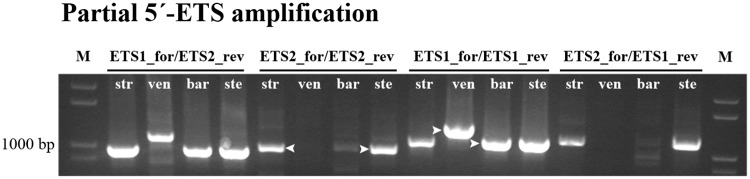
Partial 5’-ETS sequence. PCR amplification of partial 5’-ETS obtained with the following primers combinations: ETS1_for/ETS2_rev; ETS2_for/ETS2_rev; ETS1_for/ETS1_rev; and ETS2_for/ETS1_rev on *A*. *strigosa* (str); *A*. *ventricosa* (ven); *A*. *barbata* (bar) and *A*. *sterilis* (ste). M: molecular marker 1Kb+. Arrowheads indicate bands sequenced.

We started our study of the 45S rDNA 5’ external transcribed spacer (5’-ETS) amplifying a region between TIS sequence (TATAGTAGGG) and the beginning of D repeat using two pairs of primers (Table 1) to amplify fragments with the following expected sizes: ETS1_for/ETS1_rev—1089 bp; ETS2_for/ETS2_rev—977 bp; ETS1_for/ETS2_rev—942 bp and ETS2_for/ETS1_rev—1129 bp. The results presented in Fig 3 show that the four primers combinations yielded bands with the expected dimension in species with the A-genome—*A*. *strigosa*, *A*. *barbata* and *A*. *sterilis*, although *A*. *barbata* bands obtained with the primer ETS2_for showed a considerable lower intensity. On the other hand, the C genome diploid species *A*. *ventricosa* yielded a larger band (more ~150 bp) with primer ETS1_for and a lack of amplification products with primers ETS2_for. *A*. *sativa* produced bands with the expected sizes as well as the other species with A genome. *A*. *eriantha* results were similar to the ones described for *A*. *ventricosa* (results not shown). So it seems that the ETS2_for sequence is absent in the C-genome diploid species.

**Fig 4:**
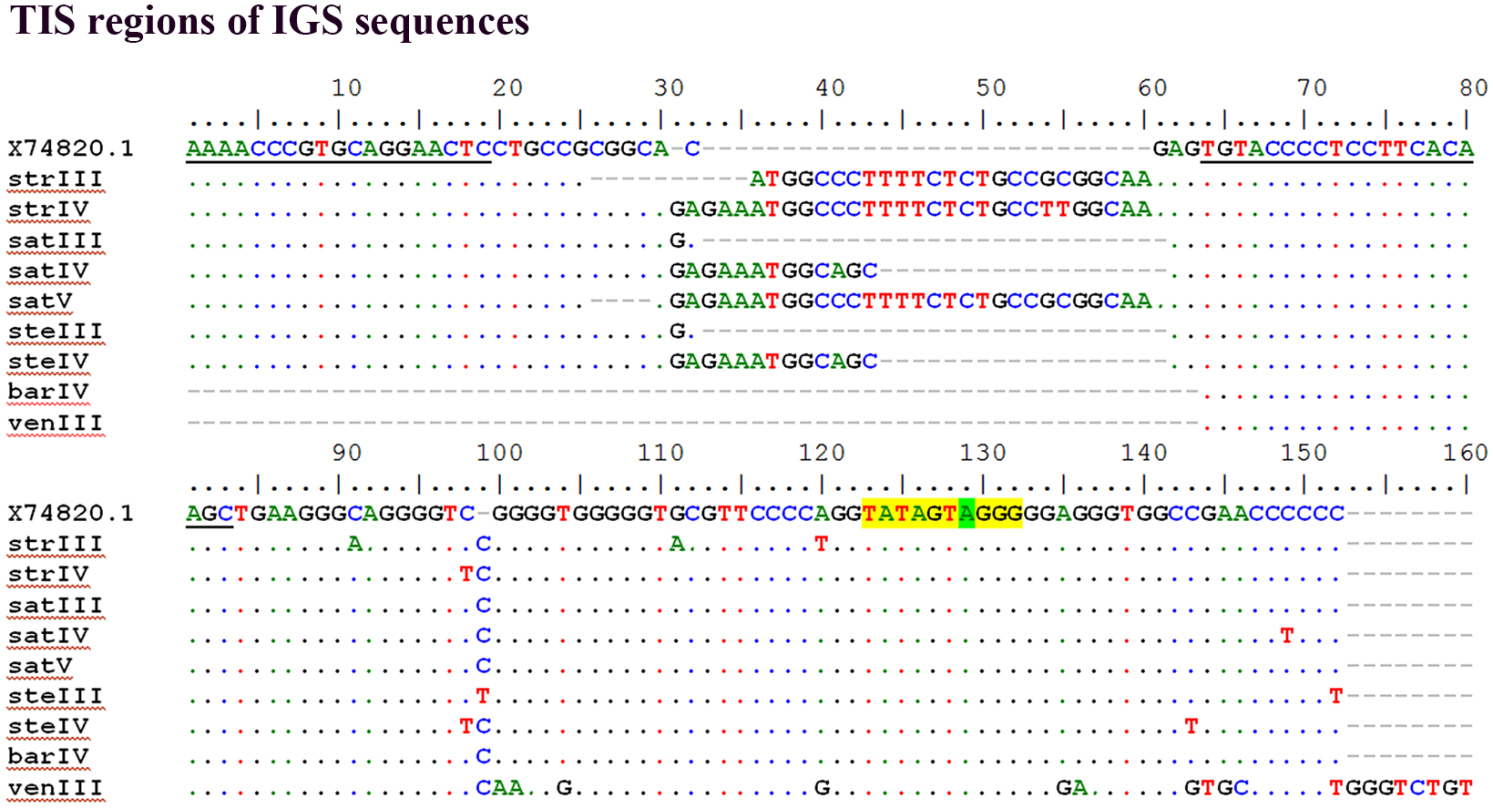
TIS regions of IGS sequence. Alignment of IGS sequences surrounding the transcription initiation site (TIS) from *A*. *strigosa* (str), *A*. *sativa* (sat), *A*. *sterilis* (ste), *A*. *barbata* (bar), *A*. *ventricosa* (ven) with *A*. *sativa* published sequence (Accession Number: X74820.1). Roman numerals indicate different clones. Primer sequence of ETS1_for (from position 64 to 83) and ETS2_for (from position 1 to 19) are underlined. TIS is highlighted in yellow. TIS position +1 is highlighted in green.

PCR amplification products obtained with primers ETS2_for/ETS2_rev in *A*. *strigosa*, *A*. *sativa* and *A*. *sterilis* and with primers ETS1_for/ETS1_rev in *A*. *ventricosa* and *A*. *barbata* were sequenced (arrowheads in Fig 3). The overall similarity of all sequences with the *A*. *sativa* published sequence was 94 to 99% except in *A*. *ventricosa* which shows 66% similarity traduced by unexpected E repeats in the 507-881bp region and only one C repeat. Additionally, one shorter clone from *A*. *sativa* revealed the lack of the C2 repeat. The analysis of the region surrounding TIS (Fig 4) revealed insertions of a 30bp sequence or fragments of it, in *A*. *strigosa*, *A*. *sativa* and *A*. *sterilis*. A search for regulatory elements in these sequences performed in PLACE database [39] revealed that most motifs detected were related to promoter sequences. Moreover, this study unraveled variability around TIS namely in the RNA polymerase I recognized site established in several plant species (reviewed in [40]) in *A*. *ventricosa* and even a truncated shorter variant lacking TIS was observed in *A*. *sterilis*.

**Fig 5:**
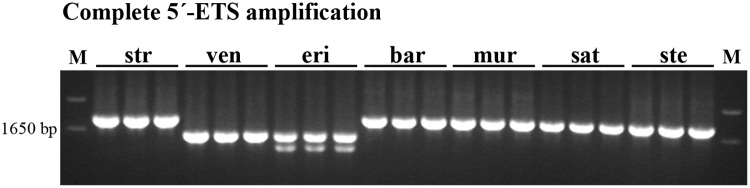
Complete 5’-ETS sequence. PCR amplification of the complete 5’-ETS obtained with primer combination ETS1_for/18S from *A*. *strigosa* (str); *A*. *ventricosa* (ven); *A*. *eriantha* (eri); *A*. *barbata* (bar); *A*. *murphyi* (mur); *A*. *sativa* (sat) and *A*. *sterilis* (ste). M: molecular marker 1Kb+.

Since the amplification using ETS1_for primer was effective in all genotypes analyzed, it was further used combined with 18S primer to amplify 5’-ETS complete sequences in all species studied. The results obtained presented in Fig 5 disclosed bands with similar length (~1750bp) in all species with A-genome–*A*. *strigosa*, *A*. *barbata*, *A*. *murphyi*, *A*. *sativa* and *A*. *sterilis* while C-genome diploid species–*A*. *ventricosa* and *A*. *eriantha*–showed shorter 5’-ETS sequences (~1500bp) and *A*. *eriantha* revealed an additional band with 1400bp.

Complete 5’-ETSs were sequenced using internal primers based on the partial sequences previously referred (primers ETS3_for and ETS3_rev, Table 1). Their alignment along with the partial sequences is presented in S2 Fig. Sequence similarity analysis (summarized in Table 2) revealed 96 to 99% homology amongst species with A genome and 99% similarity in C-genome diploid species. The similarity observed between species with A- and diploid species with C-genome was considerably lower (ranging from 70 to 76%) and few SNPs were detected upstream the 18S, a region prone to loops formation. However, these SNPs do not appear to affect loop conformation significantly since Mfold [41] analysis revealed similar structures with similar free energies (-29.90 kcal/mol in species with A-genome and -30.50 kcal/mol in C-genome diploid species).

**Table 2:** Similarity between complete 5’-ETS sequences of all *Avena* species analyzed.

**%**	**X74820**	**Str**	**Ven**	**EriL**	**EriS**	**Bar**	**Mur**	**Sat**	**Ste**
**X74820**	100	97	70	71	75	97	98	96–97	96–99
**Str**		100	71	71	75	99	98	99	98
**Ven**			100	99	99	71–72	71	71–72	71–72
**EriL**				100	100	71	70–71	71–72	71
**EriS**					100	75–76	75	75–76	75–76
**Bar**						98	98	98–99	98–99
**Mur**							100	98	98
**Sat**								100	98–100
**Ste**									98

Notes:

EriL and EriS represent the sequences of the larger and shorter bands of *A*. *eriantha*, respectively.

The similarity between the sequences of species with A-genome and C-genome diploid species is highlighted in light grey.

**Fig 6:**
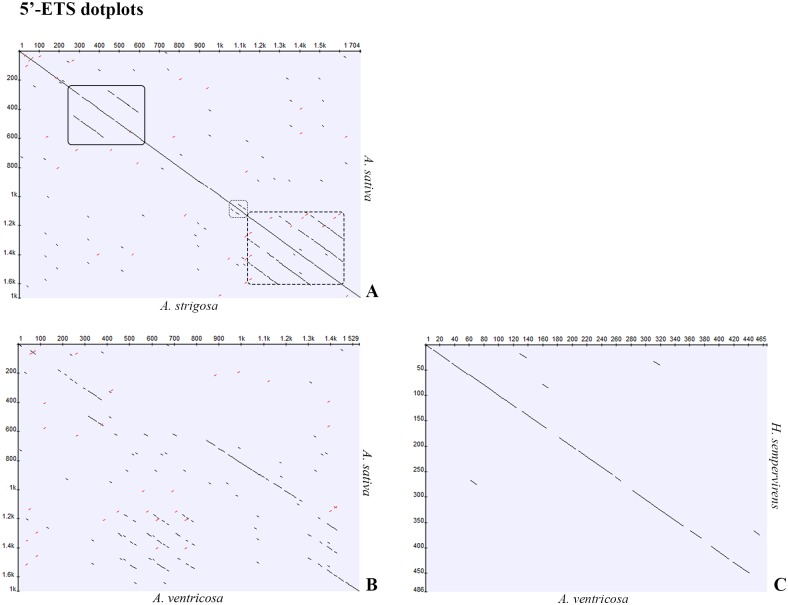
5’-ETS sequence dotplots. Dotplots (min length 8, identity 100%) of the 5’-ETS sequences in comparison between the published sequence of *A*. *sativa* (Accession Number: X74820.1) and **A)** *A*. *strigosa* (Accession Number: KM586737) or **B)** *A*. *ventricosa* (Accession Number: KM586759). **C)** Comparison of the +1011 to +1475 region of *A*. *ventricosa* with the homologous *H*. *sempervirens* sequence (Accession Number: GQ324269.1). Solid underline highlights the C repeats, dot outlines D repeats and dash outline highlights the E repeats.

**Fig 7:**
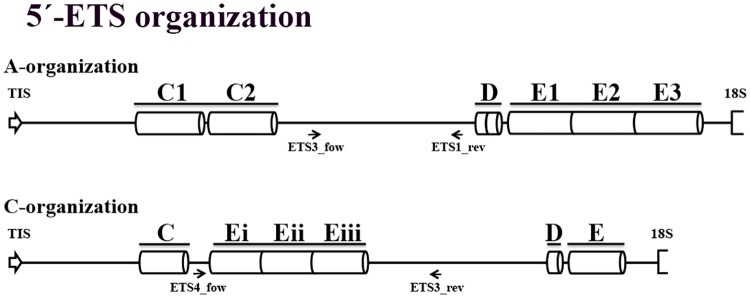
5’-ETS organization types. Representation of 45S rDNA 5’-ETS organization in species with A-genome (A-organization) and in diploid species with C-genome (C-organization). Arrows represent the relative location of the internal primers used to sequence the complete 5’-ETS.

To identify repetitive elements and further understand the organization of the 5’-ETS, dotplots were constructed. Given the high similarity observed between all species with A-genome studied, the *A*. *strigosa* sequence was used to construct a dotplot in comparison with *A*. *sativa* published sequence (Fig 6A) revealing the repeats previously reported: two C repeats with ~150bp in the 300-600bp region; two short D repeats in the 1100–1200 bp region; and three E repeats with approximately 120 bp in the 1200-1600bp region. This 5’-ETS organization is henceforward referred as A-organization (Fig 7).

Since the ~1500bp sequences of C-genome diploid species—*A*. *ventricosa* and *A*. *eriantha*—are similar, the *A*. *ventricosa* sequence was selected to construct a dotplot along with the *A*. *sativa* published sequence (Fig 6B). C-genome diploid species dotplot analysis revealed the presence of only one C repeat, as also revealed by the partial sequences described, followed by three E repeats absent in A-organization. Additionally, only one D repeat and one E repeat are present upstream the 18S sequence. This organization described in *A*. *ventricosa* is henceforth referred as C-organization (Fig 7) and explains the unexpected presence of shorter 5’-ETS sequences in C-genome diploid species (Fig 5). In fact, *A*. *ventricosa* partial sequence is larger (Fig 3) due to the higher number of E repeats downstream C repeat but the complete sequence is shorter due to the lower number of E repeats downstream the D repeat (Fig 7). Finally, the 1400bp shorter variant unraveled in *A*. *eriantha* lacks the first copy of the E repeat.

Interspecific comparisons of E repeats show the highest similarity between *A*. *sativa* repeats E2 and E3 and the *A*. *ventricosa* repeat closest to 18S (82%). E repeats comparison within *A*. *ventricosa* revealed that the three repeats closer to the C repeat are more similar (94–96%) than the one closer to 18S (75 to 78%). Interestingly, the *A*. *ventricosa* E repeat proximal to 18S is more similar (91%) to other Aveninae sub-tribe species as *Helictotrichon* (GQ324269.1) than with the other *Avena* E repeats analyzed. *A*. *sativa* E2 and E3 repeats present 81% similarity with the *Helictotrichon* sequence.

### Unraveling *Avena* A and C genomes evolutive clues

**Fig 8:**
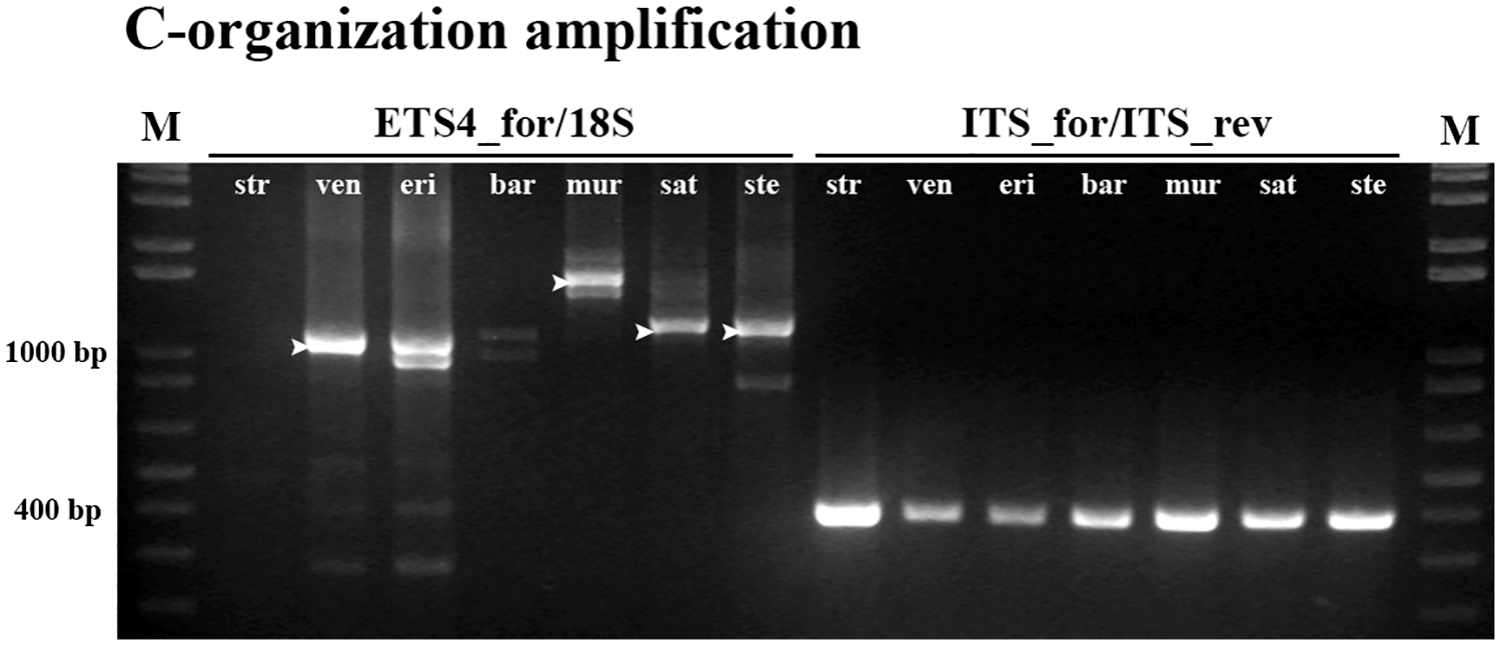
C-organization specific amplification. PCR amplification obtained with primers ETS4_for/18S (2–8) and primers ITS2_for/ITS2_rev (9–15); 16 –molecular marker 1Kb+. *A*. *strigosa* (str); *A*. *ventricosa* (ven); *A*. *eriantha* (eri); *A*. *barbata* (bar); *A*. *murphyi* (mur); *A*. *sativa* (sat) and *A*. *sterilis* (ste). M: molecular marker 1Kb+. Arrowheads mark the bands isolated for sequence analysis.

To assess the presence of sequences with C-organization in polyploid species with C-genome a ETS4_for primer (for localization see Fig 7) was designed based on our *A*. *ventricosa* partial sequences (Accession Number: KM586761) and combined with 18S primer (Fig 8, ETS4_for/18S). As control 45S rDNA ITS2 sequence was amplified yielding the expected band with ~380bp in all species (Fig 8, ITS_for/ITS_rev). ETS4_for primer matches an IGS sequence domain downstream the C repeat in *A*. *ventricosa* that is absent in the *A*. *sativa* sequence (Fig 7) and the absence of amplification in A-genome diploid *A*. *strigosa* confirms that such sequence is not present in the A genome diploid species. Conversely, the amplification of *A*. *ventricosa* and *A*. *eriantha* with primers ETS4_for and 18S yielded the expected band with ~1100bp and an additional band with ~1000bp in *A*. *eriantha*. The same experiment amplified fragments with higher dimensions in polyploid species as *A*. *murphyi*, *A*. *sativa* and *A*. *sterilis*. Sequence comparative analysis of the bands amplified from the polyploid genotypes and from *A*. *ventricosa* (indicated in Fig 8) confirmed the presence of three copies of the E repeat downstream the C repeat in *A*. *ventricosa* and sequences with the C-organization type were also detected in polyploid species although with higher number of E repeats. In fact, *A*. *sativa* and *A*. *sterilis* present four repeats and in *A*. *murphyi* four, six and seven E repeats were observed.

To attempt a deeper understanding on 5’-ETS organization evolutive patterns, organization types A and C were compared with related species of the same tribe (*Puccinellia bruggemannii* Accession Number: GQ283217.1), of the same sub-tribe (*Helictotrichon sempervirens*, Accession Number: GQ324269.1); and of the same sub-family (*Triticum aestivum* Accession Number: X07841.1). This analysis revealed a higher overall homology of C-organization with sequences from *A*. *ventricosa*, *H*. *sempervirens*, *P*. *bruggemannii* and *T*. *aestivum* than between A-organization and sequences from referred related species. This great homology of C-organization with IGSs from species of other genus was moreover confirmed through dotplot matrix comparing *A*. *ventricosa* and *H*. *sempervirens* sequences (Fig 6C).

**Fig 9:**
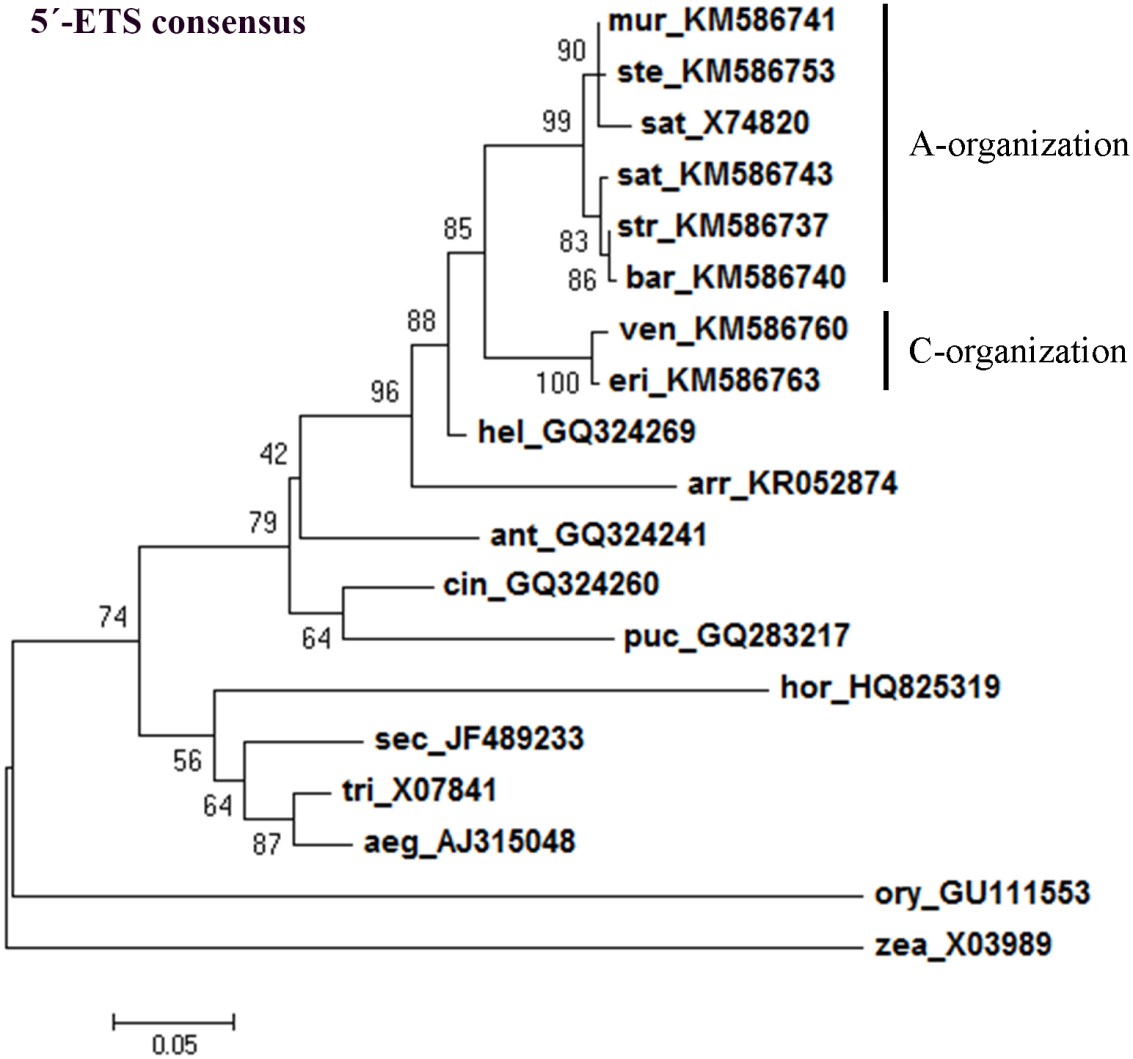
5’-ETS consensus tree. Maximum-likelihood consensus tree based on 5’-ETS from *A*. *strigosa* (str, KM586737), *A*. *ventricosa* (ven, KM586760), *A*. *eriantha* (eri, KM586763), *A*. *barbata* (bar, KM586740), *A*. *murphyi* (mur, KM586741), *A*. *sativa* (sat, KM586743) and *A*. *sterilis* (ste, KM586753); and from sequences previously published: *A*. *sativa* (sat, X74820), *H*. *sempervirens* (hel, GQ324269); *A*. *elatius* (arr, KR052874); *A*. *monticola* (ant, GQ324241), *P*. *bruggemannii* (puc, GQ283217), *C*. *arundinacea* (cin, GQ324260), *H*. *vulgare* (hor, HQ825319), *S*. *cereale* (sec, JF489233), *T*. *aestivum* (tri, X07841), *A*. *umbellulata* (aeg, AJ315048), *Z*. *mays* (zea, X03989) and *O*. *sativa* (ory, GU111553). Accession Numbers in brackets. Tree obtained using the Hasegawa-Kishino-Yano model [42] with discrete gamma distribution; numbers on the branches represent bootstrap support for 1000 replicates; scale indicates the percentage of divergence.

**Fig 10:**
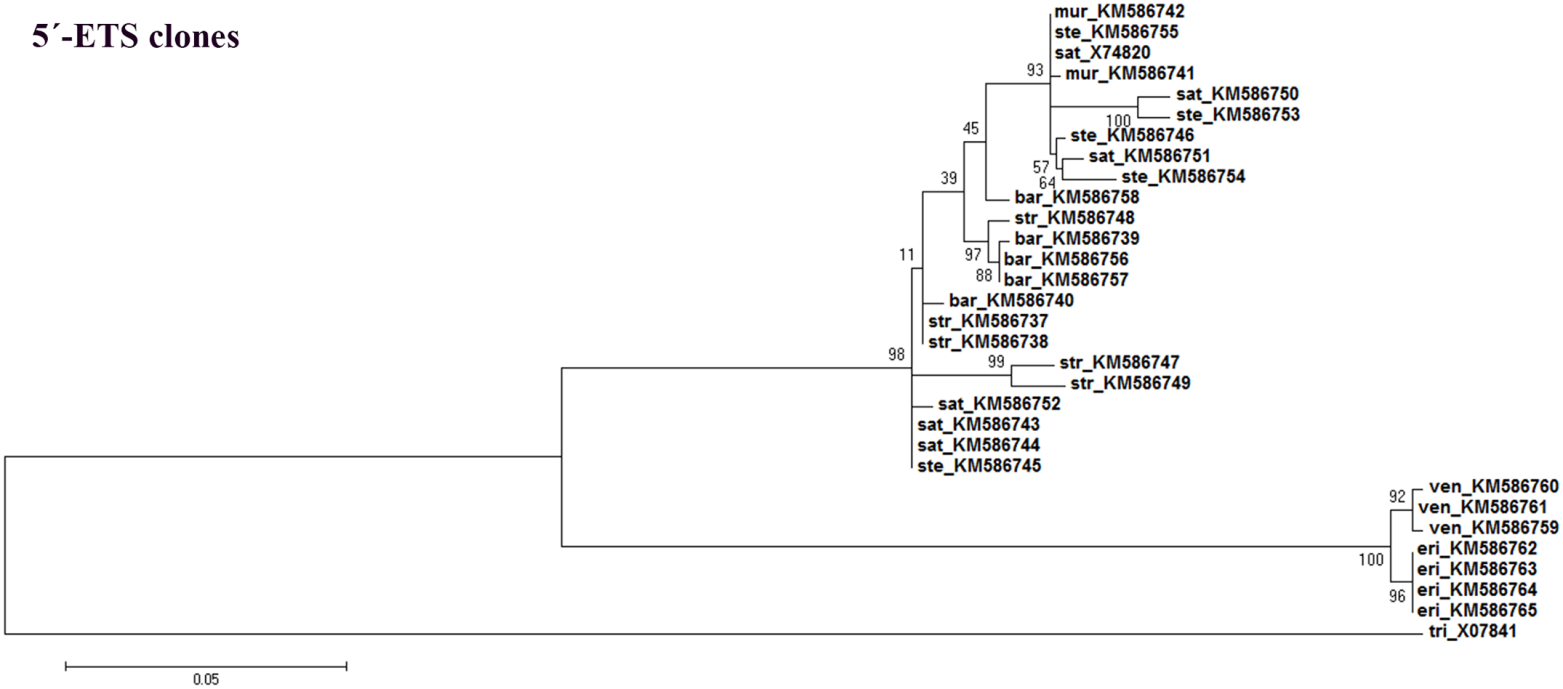
5’-ETS clones consensus tree. Maximum-likelihood consensus tree based on all complete and partial 5’-ETS sequence clones from *Avena* species analyzed (Accession Numbers in S1 Table) and from *T*. *aestivum* published sequence (Accession Number: X07841) as out-group. Tree obtained using the Kimura phylogenetic model [43]; numbers on the branches represent bootstrap support for 1000 replicates, scale indicates the percentage of divergence.

The maximum-likelihood consensus tree constructed using *Avena* 5’-ETSs obtained in this study and sequences from other Poaceae species allowed the separation of a clade comprising all *Avena* species. *Avena* species are further separated in different clades, one including C genome diploid species and the other including species with the A genome (Fig 9). Likewise, the consensus tree constructed using all clones of complete and partial 5’-ETS sequences obtained from all *Avena* genotypes analyzed (Fig 10) confirms a clear separation of C genome diploid species.

**Fig 11:**
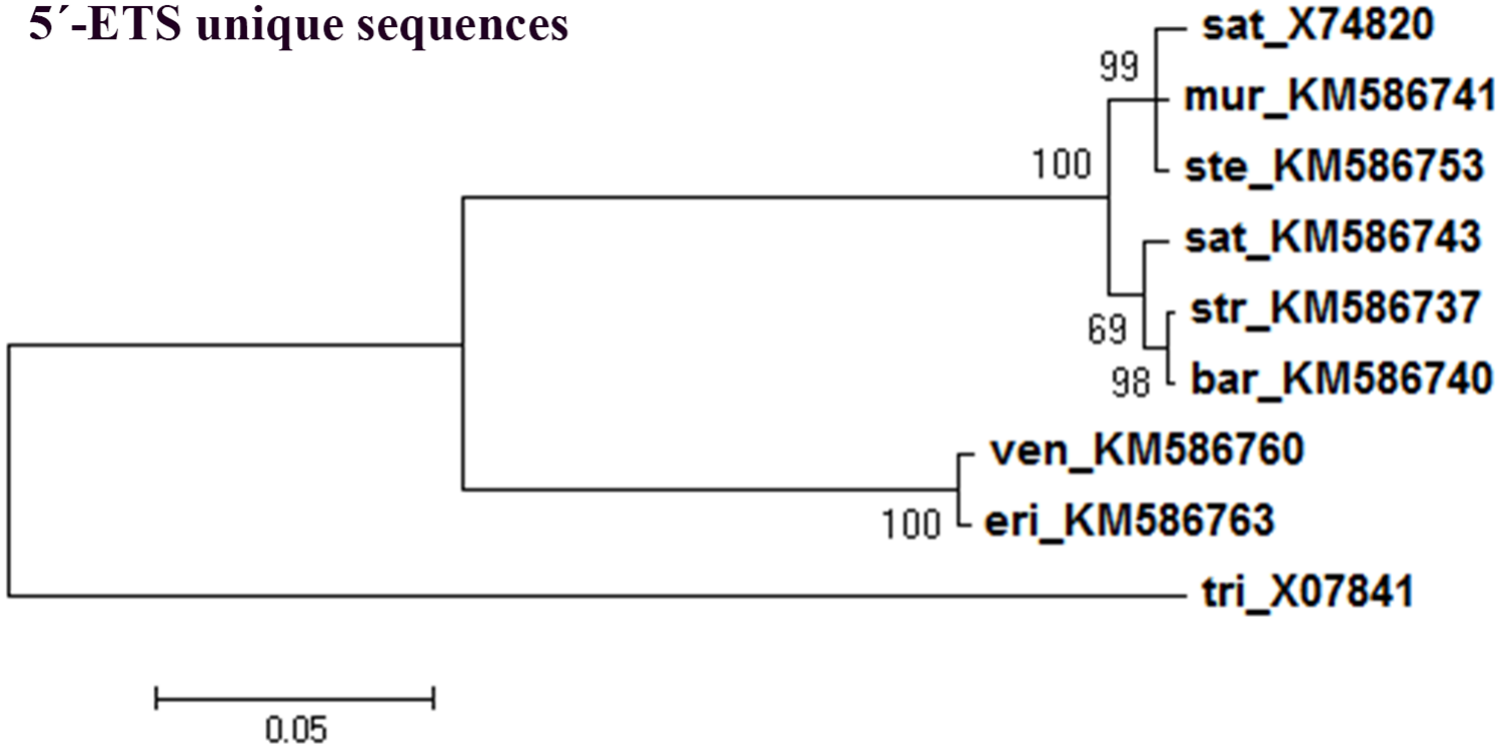
5’-ETS unique sequences consensus tree. Maximum-likelihood consensus tree based on 5’-ETS unique sequences from *Avena* (excluding repeat sequences C2, E2, E3, Ei, Eii and Eiii): *A*. *strigosa* (str, KM586737); *A*. *ventricosa* (ven, KM586760); *A*. *eriantha* (eri, KM586763); *A*. *barbata* (bar, KM586740); *A*. *murphyi* (mur, KM586741); *A*. *sativa* (sat, KM586743) and *A*. *sterilis* (ste, KM586753)and *T*. *aestivum* (tri, X07841) as the out-group. Accession Numbers in brackets. Tree obtained using the Tamura model [44]; numbers on the branches represent bootstrap support for 1000 replicates, scale indicates the percentage of divergence.

To evaluate if the phylogenetic inference described only reflects repeats organization, a phylogenetic tree was built using the external transcribed sequences excluding all repeat motifs (repeats: C2, E2, E3, Ei, Eii and Eiii when applicable). As can be seen in Fig 11, a clear separation is similarly obtained between C genome diploid species and species with A genome considering only 5’-ETS unique sequences. Moreover, clusters within the A genome branch correspond to the ones obtained with the complete 5’-ETSs (Fig 9) were observed, namely one cluster grouping *A*. *murphyi*, *A*. *sterilis* and the published *A*. *sativa* sequence and the other cluster comprising *A*. *strigosa*, *A*. *barbata* and *A*. *sativa*.

To assess the robustness of the phylogenetic trees presented in this study, sequence alignment was tested using different methods (Clustal Omega, MUSCLE, MAFFT and T-COFFEE). The evolutionary models for the alignments generated were tested in JModelTest2 and MEGA6 and the best model was selected based on the lowest BIC (Bayesian Information Criterion) score. For all models selected, phylogenetic trees were obtained using MEGA6 and in MrBayes. Overall, all trees generated clustered the species similarly reinforcing the use of the 5’-ETS for *Avena* phylogeny.

## Discussion

In this study 45S rDNA IGS sequences, particularly the 5’-ETS organization, were for the first time comparatively characterized in species of the genus *Avena* with different ploidy levels and genomic constitutions. The evaluation of the intergenic spacer (IGS) unraveled clear length variability between the *Avena* species *A*. *strigosa*, *A*. *barbata*, *A*. *murphyi*, *ventricosa*, *A*. *eriantha*, *A*. *sativa* and *A*. *sterilis*. IGS variability was previously reported in Poaceae between different species [45, 46] and varieties [47] as well as between distinct *A*. *sativa* accessions [27]. Additionally, besides the IGS sequences with the expected size (~4000bp) amplicons with distinct lengths were also detected in species with A genome—*A*. *strigosa*, *A*. *barbata*, *A*. *murphyi*, *A*. *sativa* and *A*. *sterilis*. Similar within species IGS length variation was also described in *A*. *sativa* [26] and more recently in *Medicago arborea* [48] and in the Fagaceae family [31]. Moreover, the detection of IGS sequences with different lengths within *Avena* species are mainly due to differences in the non-transcribed spacer since 5’-ETS amplifications yielded a unique fragment in most species, except in diploid C-genome species.

**Fig 12:**
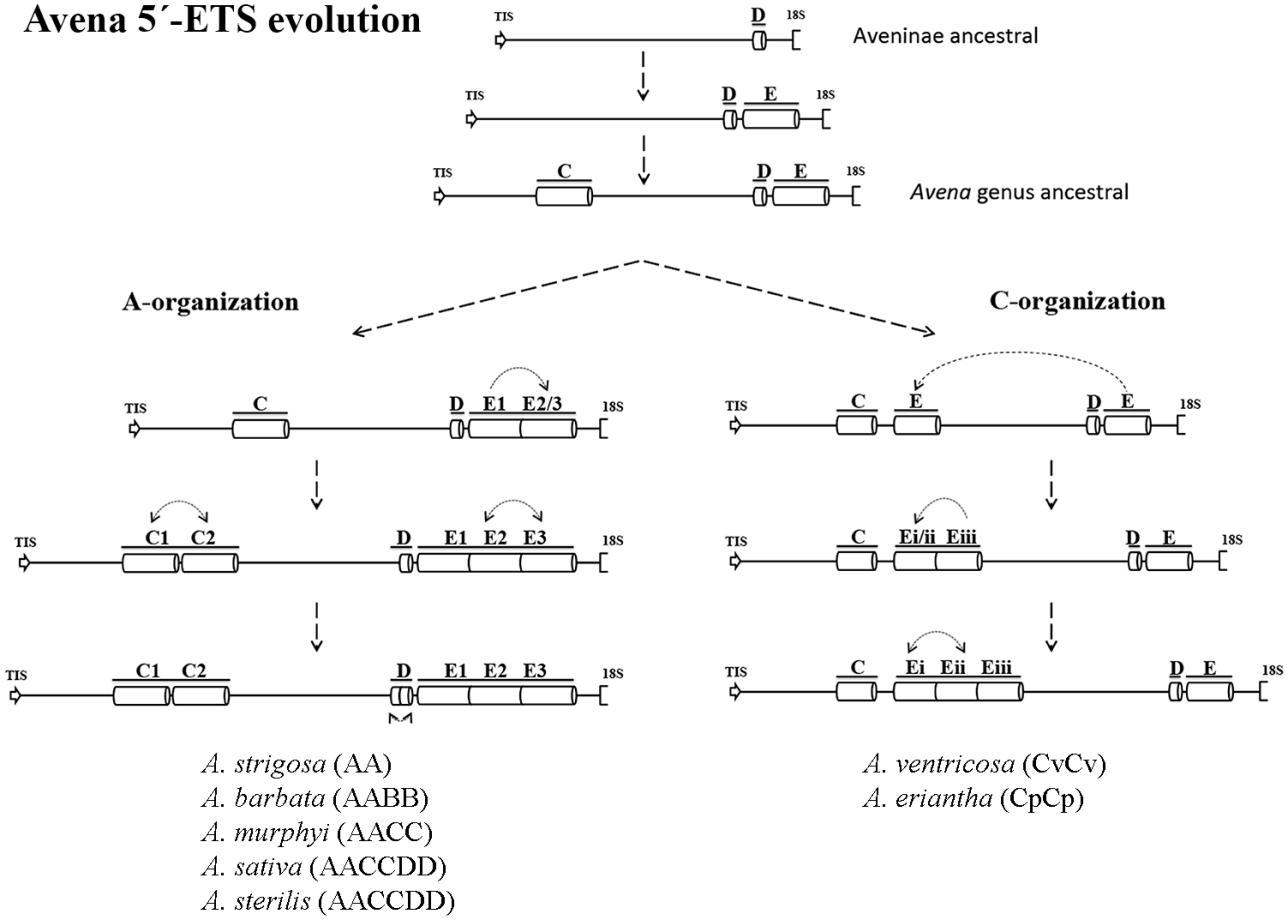
*Avena* 5’-ETS evolution. Representation of the proposed succession of events leading to the establishment of 45S rDNA 5’-ETS A- and C-organization.

The detailed analysis of the 5’-ETS sequences presented in this work shows that all species with A-genome have the repeats pattern previously described in *A*. *sativa* [26] and here designated as A-organization. However, we identified a novel 5’-ETS organization pattern in C-genome diploid species—*A*. *ventricosa* and *A*. *eriantha* -, nominated C-organization. This C-organization lacks one C and one D repeat and is also characterized by the presence of only one E repeat downstream the D repeat and two or three E repeats located between the C and D motifs (Fig 12). Both dotplot (Fig 6B and 6C) and phylogenetic tree (Fig 11) analyses of *Avena* 5’-ETS sequences in comparison with other Poaceae taxa shows a higher similarity of C-genome organization than of A-organization with those related genera, suggesting an ancestrality of the C-genome.

Additionally, the exploitation of 5’-ETS repeats diversity allows the proposal presented in Fig 12 for the succession of events involved in *Avena* genomes evolution. Comparative analysis of A and C 5’-ETS organizations with sequences from related species revealed that E repeat is only absent in *Z*. *mays* and *O*. *Sativa*, while D repeat is found in all related species analyzed (for species and Accession Numbers see Fig 9), suggesting that D repeats are the more ancestral ones. On the other hand, C repeats present in both organization types seem to be specific of *Avena* genus since apart from A. *sativa* published sequence, no similar sequences are found through BLAST in NCBI database. Thus, the differences unraveled between A- and C-organizations must have emerged after the divergence of the *Avena* genus from the ancestral Aveninae. Further considering the nucleotide polymorphisms observed between different repeats and between repeats from the distinct species analyzed (S2 Fig) we suggest that the more ancient event that occurred in A-organization was E repeat duplication, followed by C repeat duplication and a second duplication of the E repeat and, lastly, a D repeat duplication arose. Considering the organization pattern disclosed in C genome diploid species, the first plausible event was the duplication/translocation of the final E repeat to upstream the D repeat, followed by subsequent duplications of E repeats that seem to occurred more recently.

Our results prove that the 45S rDNA 5’-ETS domain can be a particularly valuable option to enlighten the phylogenetic pathways of the genus *Avena*. Indeed, the tree presented based on the 5’-ETS consensus sequences unraveled a clear separation between the C-genome diploid species and the other *Avena* species. It must moreover be emphasized that those clusters of *Avena* species are revealed both by the analysis of entire 5’-ETS sequences (Fig 9) as well as when repeat motifs are excluded (Fig 11). Thus, besides the described 5’-ETS repeats narrative, this IGS region discloses a consistent evolutionary history since the phylogenetic analysis performed suggest that both 5’-ETS unique sequences and repeat motifs were under the same selection force.

This is particularly important considering the high homology of the internal transcribed spaces traditionally used in phylogenetic studies extensively reported in *Avena* [5, 12, 23–25]. Concordantly, 5’-ETS sequences have been previously considered more efficient than traditional ITS1 and ITS2 to study phylogenetic relationships between related species [31, 49].

The present work may also be relevant in the context of rDNA *loci* remodeling events induced by polyploidization [50, 51] corroborating that sequences from C-genome origin NORs tend to be eliminated in *Avena* polyploid species [5] since complete 5’-ETS C-organization was not detected in polyploid species with C genome. However, the detection of partial C-genome 5’-ETS sequences in polyploid species *A*. *murphyi*, *A*. *sativa* and *A*. *sterilis* using C-organization specific primers suggests the occurrence of complex restructuring events induced by polyploidization. Similarly, molecular traces of C-genome specific ITS sequences were reported in polyploid species by [5] corresponding to 1.5% of C-genome origin rDNA copies. Furthermore, it can be suggested that C-genome sequences loss may be correlated with differences in parental genome size in *Avena* polyploid species. Indeed, C genome is larger (5.48 pg *A*.*ventricosa*) than A-genome (4 to 5.33 pg in A-genome diploid species) (Plant DNA C-values Database, [52]). Thus, C-organization 5’-ETS sequence loss may be a part of parental genome size homogenization that may preferentially affects the larger parental genome to stabilize *Avena* polyploid species, as proposed to other *Poaceae* polyploid species [53]. An integrative view of the data obtained in this study evidences that the study of *Avena* ribosomal DNA evolution patterns constitute an interesting model to assess Poaceae taxa evolutionary pathways as well as to understand restructuring events associated with polyploidization.

## Supporting information

S1 Table Accession numbers. Accession numbers of *Avena* sp. 45S rDNA intergenic sequences.
(PDF)

S1 Fig *A*. *sterilis* shorter IGS. Consensus sequence of *A*. *sterilis* shorter intergenic spacer sequence.
(PDF)

S2 Fig *Avena* sp. 5’ ETS sequences. Alignment of complete and partial *Avena* sp. 5’ ETS sequences with the published sequence of *A*. *sativa* between 2320 and 4098 bp (Accession Number: X74820.1). Complete sequences obtained from *Avena strigosa*, *A*. *barbata*, *A*. *murphyi*, *A*. *sativa and A*. *stetilis* (strI to steII), partial sequences obtained with primer ETS2_for from *A*. *strigosa*, *A*. *sativa and A*. *sterilis* (strIII to steV) and with primer ETS1_for from *A*. *barbata*, *A*. *ventricosa and A*. *eriantha* (barIII to eriIV).
(PDF)
