# Supplementary material for: 45S rDNA external transcribed spacer organization reveals new phylogenetic relationships in Avena genus
Source: PLoS One. 2017 Apr 27;12(4):e0176170. doi: 10.1371/journal.pone.0176170 (PMC5407837; doi:10.1371/journal.pone.0176170)
Supplement: S1 Table — Accession numbers of Avena sp. 45S rDNA intergenic sequences. (PDF) [file pone.0176170.s001.pdf]

S1 Table. **Accession numbers**. Accession numbers of *Avena* sp. 45S rDNA intergenic sequences.

**Clone Description**	**Accession Number**
Avena strigosa, 45S rDNA external transcribed spacer, complete sequence, clone I	KM586737
Avena strigosa, 45S rDNA external transcribed spacer, complete sequence, clone II	KM586738
Avena strigosa, 45S rDNA external transcribed spacer, partial sequence, clone III	KM586747
Avena strigosa, 45S rDNA external transcribed spacer, partial sequence, clone IV	KM586748
Avena strigosa, 45S rDNA external transcribed spacer, partial sequence, clone V	KM586749
Avena ventricosa, 45S rDNA external transcribed spacer, complete sequence, clone I	KM586759
Avena ventricosa, 45S rDNA external transcribed spacer, complete sequence, clone II	KM586760
Avena ventricosa, 45S rDNA external transcribed spacer, partial sequence, clone III	KM586761
Avena ventricosa, 45S rDNA external transcribed spacer, partial sequence, clone IV	KM586772
Avena ventricosa, 45S rDNA external transcribed spacer, partial sequence, clone V	KM586773
Avena eriantha, 45S rDNA external transcribed spacer, complete sequence, clone I	KM586762
Avena eriantha, 45S rDNA external transcribed spacer, complete sequence, clone II	KM586763
Avena eriantha, 45S rDNA external transcribed spacer, complete sequence, clone III	KM586764
Avena eriantha, 45S rDNA external transcribed spacer, complete sequence, clone IV	KM586765
Avena barbata, 45S rDNA external transcribed spacer, complete sequence, clone I	KM586739
Avena barbata, 45S rDNA external transcribed spacer, complete sequence, clone II	KM586740
Avena barbata, 45S rDNA external transcribed spacer, partial sequence, clone III	KM586756
Avena barbata, 45S rDNA external transcribed spacer, partial sequence, clone IV	KM586757
Avena barbata, 45S rDNA external transcribed spacer, partial sequence, clone V	KM586758
Avena murphyi, 45S rDNA external transcribed spacer, complete sequence, clone I	KM586741
Avena murphyi, 45S rDNA external transcribed spacer, complete sequence, clone II	KM586742
Avena murphyi, 45S rDNA external transcribed spacer, partial sequence, clone III	KM586766
Avena murphyi, 45S rDNA external transcribed spacer, partial sequence, clone IV	KM586767
Avena murphyi, 45S rDNA external transcribed spacer, partial sequence, clone V	KM586768
Avena sativa, 45S rDNA external transcribed spacer, complete sequence, clone I	KM586743
Avena sativa, 45S rDNA external transcribed spacer, complete sequence, clone II	KM586744
Avena sativa, 45S rDNA external transcribed spacer, partial sequence, clone III	KM586750
Avena sativa, 45S rDNA external transcribed spacer, partial sequence, clone IV	KM586751
Avena sativa, 45S rDNA external transcribed spacer, partial sequence, clone V	KM586752
Avena sativa, 45S rDNA external transcribed spacer, partial sequence, clone VI	KM586769
Avena sativa, 45S rDNA external transcribed spacer, partial sequence, clone VII	KM586770
Avena sterilis, 45S rDNA external transcribed spacer, complete sequence, clone I	KM586745
Avena sterilis, 45S rDNA external transcribed spacer, complete sequence, clone II	KM586746
Avena sterilis, 45S rDNA external transcribed spacer, partial sequence, clone III	KM586753
Avena sterilis, 45S rDNA external transcribed spacer, partial sequence, clone IV	KM586754
Avena sterilis, 45S rDNA external transcribed spacer, partial sequence, clone V	KM586755
Avena sterilis, 45S rDNA external transcribed spacer, partial sequence, clone VI	KM586771
Avena sterilis, 45S rDNA intergenic spacer, clone VII	KM586774
Avena sterilis, 45S rDNA intergenic spacer, clone VIII	KM586775
