# Supplementary material for: 45S rDNA external transcribed spacer organization reveals new phylogenetic relationships in Avena genus
Source: PLoS One. 2017 Apr 27;12(4):e0176170. doi: 10.1371/journal.pone.0176170 (PMC5407837; doi:10.1371/journal.pone.0176170)
Supplement: S1 Fig — Consensus sequence of A. sterilis shorter intergenic spacer sequence. (PDF) [file pone.0176170.s002.pdf]

S1 Fig. ***A. sterilis* shorter IGS.** Consensus sequence of *A. sterilis* shorter intergenic spacer sequence.

```
GACGACTTAAATACGCGACGGGGCATTGTAAGTGGCAGAGTGGCCTTGCTGCCACGATCCACTGAGATCCAGCCCCACGTCGCACGGATTCGTTACCTCGTGGTACTGGCACGTTT
TGTGCTCGGTGCTATCAAGGAAGCCTCGCTCTCGCTATTGGTTTCGGATGCCGCTCACGATAATGGTTAATGGCCCTTCTGGTTGCCGCTCACGATAAAGGTTAATAGCCCTTCTG
GTTGTCGCTAGTCCCACCTGAAAAGAAGCTTTCTGGATCTGAACGGCGGTAGTACGGTGTGTTGCATGTATTTCCCACAGTTTGTGGGGAGAAGCAACACGCTTGGAAACCTCTCT
AGTAGGAAAAGGAAAGCTTAGTCCCGTTCAAGTGCGACAACCGGACCGGCTATTGACCTCAAAACARGCTCTCTGGATCAGAACGGTCTTAGTACGGGGTGTTGCATGTATTCCCC
ACATTTTGTGGTAGGAGACGCATCACGCTGGAAATGTCTCTAGTAGGAGAAGGAAAGCTTAGTCCCGCTTAATAGCGACAACCGGAACGGCTGTTGACCCACCTCAAAAGAAGCCA
TCTGGATCAGAACGGACTTAGTACGGGGTGTTGCATGTATTCCCCACAGTTTGTGGTAGGAGACGCAACACGCTGGAAATGTCTCTAGTAGGTGAAGGAAAGCTTAGTCCCGATGA
ATTGCGACAACCGGACCGGCTGTTGTCTGCCTGCAAGGGCGGATGACTACCGTCGCTGGACGTCGAAGAGGACTCGCTACCTGGTTGATCCTGCCAGTAGTCATATGCTTGTCT
```

Notes: The 810bp sequence encompasses the terminal part of 25S (underlined) and 18S (dash underlined) sequence and a region downstream of the 25S (+975 to +1656) homologous to *A. sativa* sequence (Accession Number: X74820.1). Highlighted in yellow is the nucleotide different between different sequenced clones.
