# Supplementary material for: 45S rDNA external transcribed spacer organization reveals new phylogenetic relationships in Avena genus
Source: PLoS One. 2017 Apr 27;12(4):e0176170. doi: 10.1371/journal.pone.0176170 (PMC5407837; doi:10.1371/journal.pone.0176170)
Supplement: S2 Fig — Alignment of complete and partial Avena sp. 5’ ETS sequences with the published sequence of A. sativa between 2320 and 4098 bp (Accession Number: X74820.1). Complete sequences obtained from Avena strigosa, A. barbata, A. murphyi, A. sativa and A. stetilis (strI to steII), partial sequences obtained with primer ETS2_for from A. strigosa, A. sativa and A. sterilis (strIII to steV) and with primer ETS1_for from A. barbata, A. ventricosa and A. eriantha (barIII to eriIV). (PDF) [file pone.0176170.s003.pdf]

S2 Fig. *Avena* sp. 5' ETS sequences. Alignment of complete and partial *Avena* sp. 5' ETS sequences with the published sequence of *A. sativa* between 2320 and 4098 bp (Accession Number: X74820.1). Complete sequences obtained from *Avena strigosa*, *A. barbata*, *A. murphyi*, *A. sativa* and *A. stetilis* (strI to steII), partial sequences obtained with primer ETS2_fow from *A. strigosa*, *A. sativa* and *A. sterilis* (strIII to steV) and with primer ETS1_fow from *A. barbata*, *A. ventricosa* and *A. eriantha* (barIII to eriIV). The alignment of all the sequences was obtained using the ClustalW application and manually adjusted using BioEdit Sequence Alignment Editor.

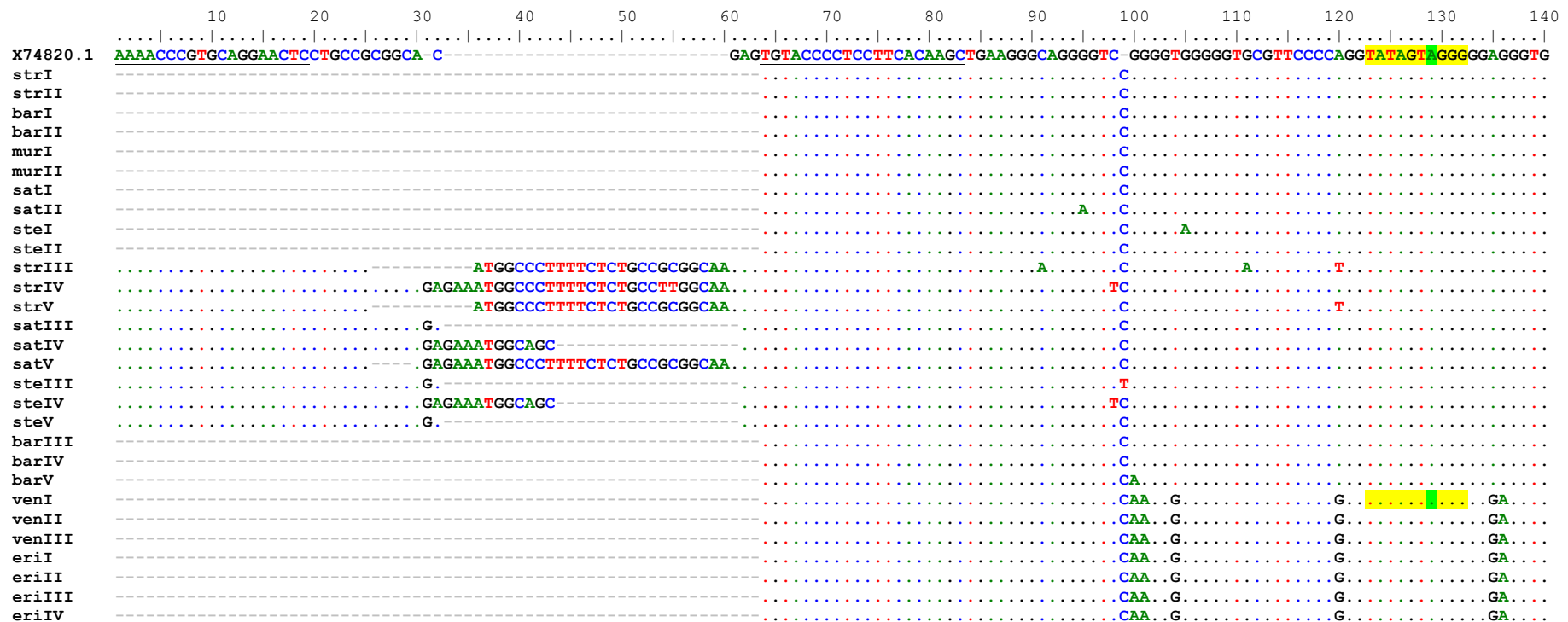

	430	440	450	460	470	480	490	500	510	520	530	540	550	560
X74820.1	CGTCCACGATACTTGGGAGCCGTTGCCCTGGTCATGTAACGTTCCAGTCGGTATGATTCGCCAAAAAGGTGATTGCCGCGTTACGTTTTTCGTGTTCCATGTGGCTGCTTCTCCTCAGTCGCTCTGGGTGGTCATCC													
strI	.....T.AC.G.C...TC.....T.....A													
strII	.....T.AC.G.C...TC.....T.....A													
barI	.A.....CGGCGT.....C.....T.....T.A													
barII	.....T.AC.G.C...TC.....T.....A.....G													
murI	.....													
murII	.....													
satI	.....T.AC.G.C...TC.....A.....A													
satII	.....T.AC.G.C...TC.....A.....A													
steI	.....T.AC.G.C...TC.....A.....A													
steII	.....T.....T.....													
strIII	...G...G...T.AC.G...TC.....A.....A.....G													
strIV	.A.....C.....T.....A.....A													
strV	...G...G...T.AC.G.C...TC.....A.....A													
satIII	.....T.....A.....													
satIV	.....A.....													
satV	.....T.AC.G.C...TC.....A.....A													
steIII	.....T.....T.....													
steIV	.....T.....C.....													
steV	.....T.....													
barIII	.A.....C.....T.....T.A													
barIV	.A.....C.....T.....T.A													
barV	.A.....C.....T.....A													
venI	.A...G...CGGCGT...T.G.TG.C...GC...T...C...T...T...A...AA...CTGT...TA.C...A.GT													
venII	.A...G...CGGCGT...T.G.TG.C...GC...T...C...T...T...A...AA...CTGT...TA.C...A.GT													
venIII	.A...G...CGGCGT...T.G.TG.C...GC...T...C...T...T...A...AA...CTGT...TA.C...A.GT													
eriI	.A...G...CGGCGT...T.G.TG.C...GC...T...C...T...T...A...AA...CTGT...TA.C...A.GT													
eriII	.A...G...CGGCGT...T.G.TG.C...GC...T...C...T...T...A...AA...CTGT...TA.C...A.GT													
eriIII	.A...G...CGGCGT...T.G.TG.C...GC...T...C...T...T...A...AA...CTGT...TA.C...A.GT													
eriIV	.A...G...CGGCGT...T.G.TG.C...GC...T...C...T...T...A...AA...CTGT...TA.C...A.GT													

	570	580	590	600	610	620	630	640	650	660	670	680	690	700
X74820.1	ATGTTAACCCAGGATAATGCGCTTGTCGGTGTCTGCCAGTACTTGGGAGCCGCTGCCCTGGTCTCGTAAACGTTCCAGTCGGTATGATTCGCCAAAAAGGTGATTGCCGCGTTACGTTTT CGTGTTCATATGGG													
strI	.....A.....T.A...G.C.....													
strII	.....A.....T.A...G.C.....													
barI	.....A.....T.A...G.C...C.....T.....T.....G													
barII	.....A.....T.A...G.C.....C.....													
murI	.....A.....T.....													
murII	.....A.....T.....													
satI	.....A.....T.A...G.C.....T.....													
satII	.....A.....T.A...G.C.....T.....													
steI	.....A.....T.A...G.C.....T.....													
steII	.....A.....T.....C...G.....													
strIII	...C.....A.....T.A...G.C.....A.....													
strIV	...C.....A.....T.A...G.C...C.....C.....													
strV	...C.....A.....T.A...G.C.....C.....A.....													
satIII	.....A.....T.....T.A.....A.....T.....													
satIV	.....													
satV	.....A.....T.A...G.C.....T.....													
steIII	.....A.....T.....A.....T.....													
steIV	.....A.....T.....C.....T.....													
steV	.....A.....A.....C...G.....A.....													
barIII	.....A.....T.A...G.C...C.....													
barIV	.....A.....T.A...G.C...C.....													
barV	.....A.....T...C.....													
venI	GGTG.TTA..T.TTCG.GC..AG.CC.TTC.AAGAAAGCTCTCCGGATCAGAAA.TGT.GTA.AA.GGTGTGTT.CCTT.TC..CAC.GTC.GTGGT.GG.TACGC.ACA.GCT.GAC..A.GCTA.ATGGGG.AGGA.A													
venII	GGTG.TTA..T.TTCG.GC..AG.CC.TTC.AAGAAAGCTCTCCGGATCAGAAA.TGT.GTA.AA.GGTGTGTT.CCTT.TC..CAC.GTC.GTGGT.GG.TACGC.ACA.GCT.GAC..A.GCTA.ATGGGG.AGGA.A													
venIII	GGTG.TTA..T.TTCG.GC..AG.CC.TTC.AAGAAAGCTCTCCGGATCAGAAA.TGT.GTA.AA.GGTGTGTT.CCTT.TC..CAC.GTC.GTGGT.GG.TACGC.ACA.GCT.GAC..A.GCTA.ATGGGG.AGGA.A													
eriI	GGTG.TTA..T.TTCG.GC..AG.CC.TTC.AAGAAAGCTCTCCGGATCAGAAA.TGT.GTA.AA.GGTGTGTT.CCTT.TCC.CAC.GTC.GTGGT.GGGTACAC.ACA.GCT.GAC..A.GCTA..AGGGG.AGGA.A													
eriII	GGTG.TTA..T.TTCG.GC..AG.CC.TTC.AAGAAAGCTCTCCGGATCAGAAA.TGT.GTA.AA.GGTGTGTT.CCTT.TCC.CAC.GTC.GTGGT.GGGTACAC.ACA.GCT.GGC..A.GCTA..AGGGG.AGGA.A													
eriIII	GGTG.TTA..T.TTCG.GC..AG.....													
eriIV	GGTG.TTA..T.TTCG.GC..AG.....													

Genomic map of the *Yersinia enterocolitica* O:4 strain 4820.1 genome, showing the location of the *Yersinia enterocolitica* O:4 strain 4820.1 genome and the location of the *Yersinia enterocolitica* O:4 strain 4820.1 genome.

The map displays the genome coordinates (1 to 840) and the corresponding DNA sequence (A, T, C, G) for the *Yersinia enterocolitica* O:4 strain 4820.1 genome. The sequence is presented in a color-coded format, with each nucleotide represented by a specific color: A (blue), T (red), C (green), and G (yellow).

The map includes the following features:

- Genome Coordinates:** The top of the map shows the genome coordinates (1 to 840) in increments of 10.
- DNA Sequence:** The sequence is presented in a color-coded format, with each nucleotide represented by a specific color: A (blue), T (red), C (green), and G (yellow).
- Gene Locations:** The map identifies the locations of several genes, including *strI*, *strII*, *barI*, *barII*, *murI*, *murII*, *satI*, *satII*, *steI*, *steII*, *strIII*, *strIV*, *strV*, *satIII*, *satIV*, *satV*, *steIII*, *steIV*, *steV*, *barIII*, *barIV*, *barV*, *venI*, *venII*, *venIII*, *eriI*, *eriII*, *eriIII*, and *eriIV*.
- Gene Structure:** The map shows the structure of the genes, including the location of the coding sequence (CDS) and the location of the regulatory elements (RE).

The map is a detailed representation of the *Yersinia enterocolitica* O:4 strain 4820.1 genome, providing a comprehensive overview of the genome structure and the location of the genes.

	990	1000	1010	1020	1030	1040	1050	1060	1070	1080	1090	1100	1110	1120
X74820.1	CGGGTAGGCACATTCGCTCGCCTTAGTCCCTTCAGA							GAAATGTTGCCCAAGACGGTATCGTTATTGGTGTAAACCCCGCCGTCGTGGACACTCA						
strI			A						T				C	
strII			A						T				C	
barI	T		A						T		C		C	
barII	A		A						T				C	
murI			A											
murII			A											
satI			A						T				C	
satII			A						T				C	TTGGT
steI			A		CATTCCATCGCCTCTAGTCCCTTCAGA				T				C	
steII	G													
strIII	T		A		TT				T	T		A	C	
strIV	T		A						T	T		C		C
strV	T	A	A	A	TT			G	T	T		A	C	
satIII			A						T				T	T
satIV	T		A											
satV			A						T				C	
steIII			A						T				T	T
steIV	T		A											
steV														
barIII	T		A						T		C		C	
barIV	T		A						T		C		C	
barV									T					
venI	ATTGTTGCCGA												CT	
venII	ATTGTTGCCGA												CT	
venIII	ATTGTTGCCGA												CT	
eriI	ATTGTTGCCGA												CT	
eriII	ATTGTTGCCGA												CT	
eriIII	ATTGTTGCCGA												CT	
eriIV	ATTGTTGCCGA												CT	

	1130	1140	1150	1160	1170	1180	1190	1200	1210	1220	1230	1240	1250	1260
X74820.1			GGACGCCCTTCGTGCAGGCCATTCCCCCGTGCTATGCGTGGGGGGAGGCTTGGTTGGCTTGACCGATGTGGATACGGCAGCGGATGAGTAGCTTTGGACCCGTCGTCTGTTGTTA											
strI														
strII														
barI									A					
barII														
murI														
murII														
satI														
satII	GTAACACCCGCGCGCGTGGACACTCA													
steI														
steII														
strIII														
strIV														
strV														
satIII														T
satIV														
satV					C	A								
steIII														
steIV														
steV														
barIII														
barIV														
barV														
venI		A	A	G	T	A		A	T	A	T	C		C
venII		A	A	G	T	A		A	T	A	T	C		C
venIII		A	A	G	T	A		A	T	A	T	C		C
eriI		A	A	G	T	A		A	T	A	T	C		C
eriII		A	A	G	T	A		A	T	A	T	C		C
eriIII		A	A	G	T	A		A	T	A	T	C		C
eriIV		A	A	G	T	A		A	T	A	T	C		C

	1270	1280	1290	1300	1310	1320	1330	1340	1350	1360	1370	1380	1390	1400
X74820.1	GATCCCCGTCCTCGTGCGGCCGACTAGCGACGCCGTGCC-GTCATTCTGTGGCTTATGTTACTGTGCTTTCAAGTGCTTGCGTGCATGTACC-GACCTACGG-AAGTGGTGCTTT-TACACGTTTGCCTCGCG-C													
strI	C.....G.....G.....C.....G.....													
strII	C.....G.....G.....C.....G.....													
barI	C.....G.....G.....C.....G.....													
barII	G.....G.....G.....C.....CG.....													
murI	C.....C.....C.....G.....T.....G.....													
murII	C.....C.....C.....G.....T.....G.....													
satI	C.....C.....G.....G.....C.....G.....													
satII	C.....C.....G.....G.....C.....G.....													
steI	C.....C.....G.....G.....C.....G.....													
steII	C.....C.....G.....T.....G.....													
strIII	G.....C.....A.....													
strIV	C.....A.....													
strV	C.....A.....													
satIII	A.....A.....CA.....													
satIV	C.....													
satV	C.....													
steIII	A.....A.....C.....C.....													
steIV	A.....C.....													
steV	C.....													
barIII	C.....C.....G.....G.....C.....G.....													
barIV	C.....C.....G.....G.....C.....G.....													
barV	C.....T.....C.....G.....G.....C.....G.....													
venI	C.....CA.....A.....C.....G.....CG.....T.....G.....													
venII	C.....CA.....A.....C.....G.....CG.....T.....G.....													
venIII	C.....CA.....A.....C.....G.....CG.....T.....G.....													
eriI	C.....CA.....C.....G.....CG.....T.....G.....													
eriII	C.....CA.....C.....G.....CG.....T.....G.....													
eriIII	C.....CA.....C.....G.....CG.....T.....G.....													
eriIV	C.....CA.....C.....G.....CG.....T.....G.....													

	1410	1420	1430	1440	1450	1460	1470	1480	1490	1500	1510	1520	1530	1540
X74820.1	GG-ACCTCTCGGTGTTCCGCTGTGGCCTAATGGCGCTTGCGGCGTTACCTCGTGGTACTGCGACGTTTCGTGCTCGGTGCTATCAAGGAAGCCTCGCTCTTGCTGTGGTTTCGGAAGCCGCACGAAAGGGTAA													
strI	AC.....G.....GC.....C.....A.....T.....													
strII	AC.....G.....GC.....C.....A.....T.....													
barI	CC.....G.....G.....GC.....C.....A.....T.....													
barII	AC.....G.....GC.....C.....A.....T.....													
murI	AC.....G.....GC.....T.....T.....													
murII	AC.....G.....GC.....T.....T.....													
satI	AC.....G.....C.....GC.....T.....C.....A.....													
satII	AC.....G.....C.....GC.....T.....C.....A.....													
steI	AC.....G.....C.....GC.....T.....C.....A.....													
steII	AC.....G.....GC.....T.....T.....													
strIII														
strIV														
strV														
satIII														
satIV														
satV														
steIII														
steIV														
steV														
barIII	CC.....G.....G.....GC.....T.....													
barIV	CC.....G.....G.....GC.....T.....													
barV	AC.....G.....GC.....C.....													
venI	AC.....TCG.....A.....T.....T.....G.....GC.....TT.....T.....A.....C.....T.....T.....A.....C.....C.....													
venII	AC.....TCG.....A.....T.....T.....G.....GC.....TT.....T.....A.....C.....T.....T.....A.....C.....C.....													
venIII	AC.....TCG.....A.....T.....T.....G.....GC.....TT.....T.....A.....C.....T.....T.....A.....C.....C.....													
eriI	AC.....TCG.....C.....A.....T.....T.....G.....GC.....TT.....T.....A.....C.....T.....T.....A.....C.....C.....													
eriII	AC.....TCG.....C.....A.....T.....T.....G.....GC.....TT.....T.....A.....C.....T.....T.....A.....C.....C.....													
eriIII	AC.....TCG.....C.....A.....T.....T.....G.....GC.....TT.....T.....A.....C.....T.....T.....A.....C.....C.....													
eriIV	AC.....TCG.....C.....A.....T.....T.....G.....GC.....TT.....T.....A.....C.....T.....T.....A.....C.....C.....													

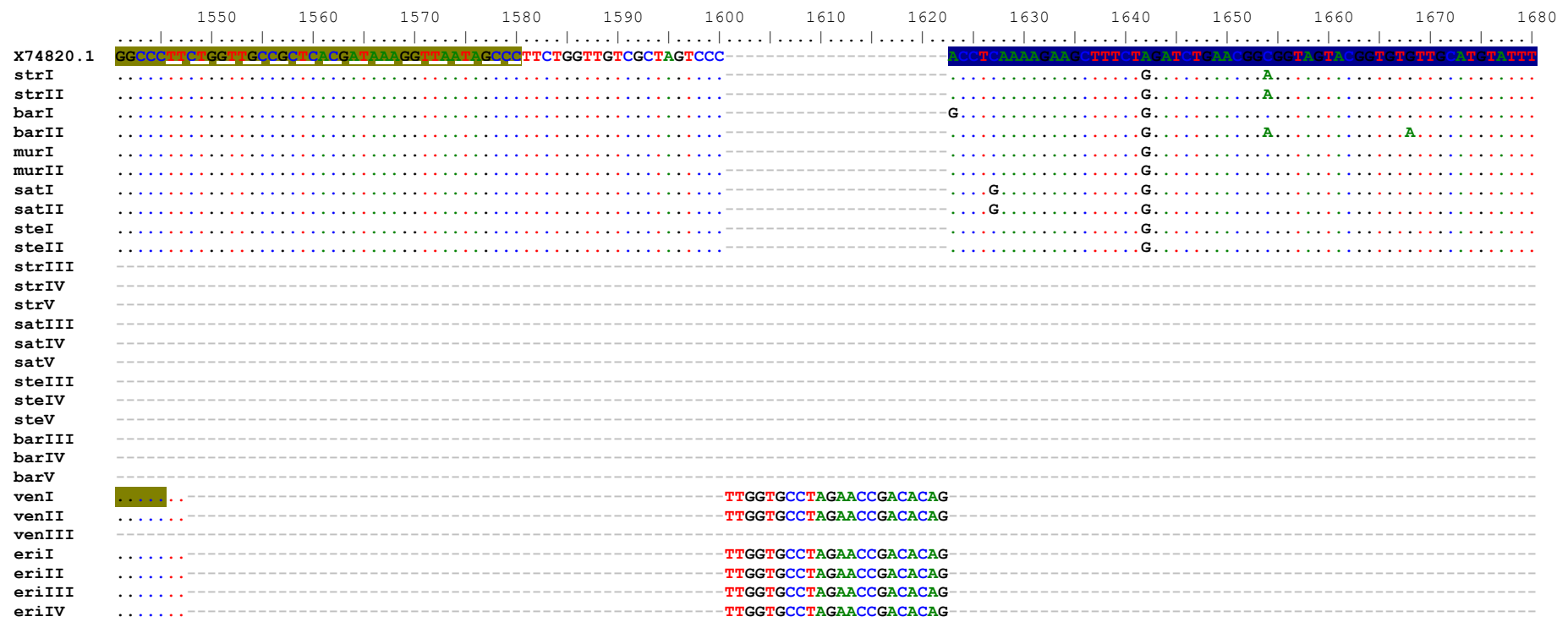

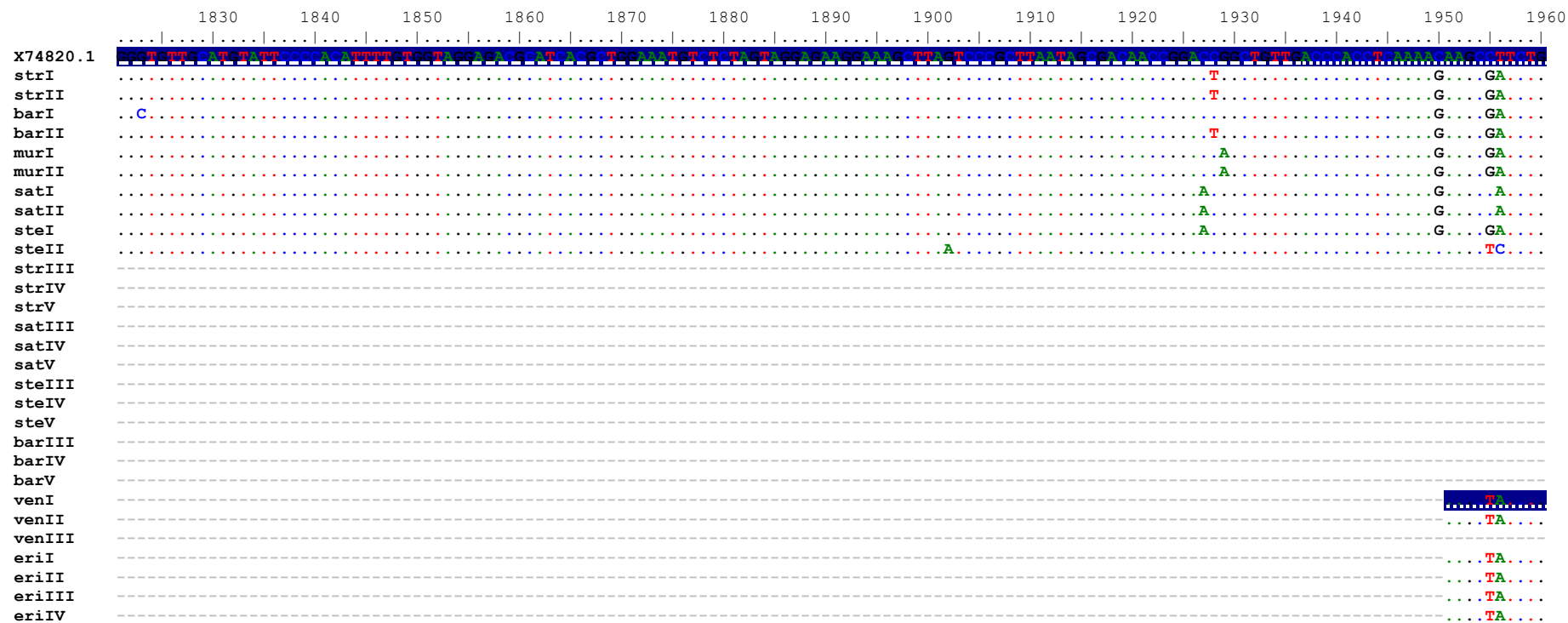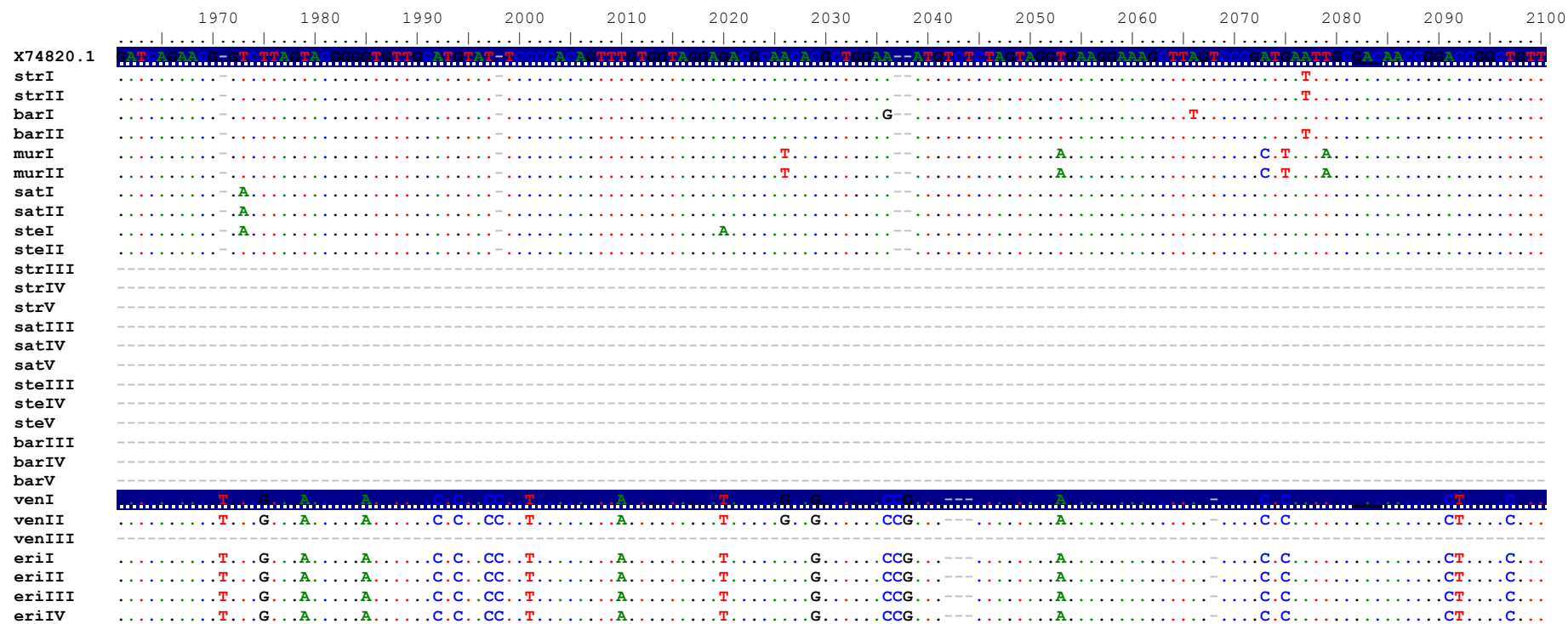

	2110	2120	2130	2140	2150	2160	2170	2180	2190
X74820.1	....								
	TCTGCCTGCAAGGGCGGATGGCTACCGTCGCTGGACGTCGAAGAGGACTCGCTACCTGGTTGATCCTGCCAGTAGTC-----								
strI	....								
strII	....								
barI	....								
barII	....								
murI	....								
murII	....								
satI	....								
satII	....								
steI	....								
steII	....								
strIII	-----								
strIV	-----								
strV	-----								
satIII	-----								
satIV	-----								
satV	-----								
steIII	-----								
steIV	-----								
steV	-----								
barIII	-----								
barIV	-----								
barV	-----								
venI	....								
venII	....								
venIII	-----								
eriI	....								
eriII	....								
eriIII	....								
eriIV	....								
